# Halophilic bacteria are colonizing the exhibition areas of the Capuchin Catacombs in Palermo, Italy

**DOI:** 10.1007/s00792-014-0649-6

**Published:** 2014-05-27

**Authors:** G. Piñar, L. Kraková, D. Pangallo, D. Piombino-Mascali, F. Maixner, A. Zink, K. Sterflinger

**Affiliations:** 1 VIBT-Vienna Institute of BioTechnology, Department of Biotechnology, University of Natural Resources and Life Sciences, Muthgasse 11, 1190 Vienna, Austria; 2 Institute of Molecular Biology, Slovak Academy of Sciences, Bratislava, Slovakia; 3 Department of Cultural Heritage and of Sicilian Identity, Sicilian Region, Palermo, Italy; 4 EURAC-Institute for Mummies and the Iceman, Bolzano, Italy

**Keywords:** Halophilic bacteria, Proteolytic capabilities, Cellulolytic capabilities, Discoloration, f-ITS, RAPD sequencing analyses

## Abstract

The Capuchin Catacombs of Palermo, Italy, contain over 1800 mummies dating from the 16th to 20th centuries AD. Their environment is not conducive to the conservation of the remains due to, among other factors, water infiltration, which is producing salt efflorescences on the walls. A multiphasic approach was applied to investigate the halophilic microbiota present in the Catacombs. Enrichment cultures were conducted on media containing different NaCl concentrations, ranging from 3 to 20 %. For screening of the strains, the following two PCR-based methods were used and compared: fluorescence internal transcribed spacer PCR (f-ITS) and random amplification of polymorphic DNA (RAPD) analyses. Results derived from RAPD profiles were shown to be slightly more discriminative than those derived from f-ITS. In addition, the proteolytic and cellulolytic abilities were screened through the use of plate assays, gelatin agar and Ostazin Brilliant Red H-3B (OBR-HEC), respectively. Many of the strains isolated from the wall samples displayed proteolytic activities, such as all strains belonging to the genera *Bacillus*, *Virgibacillus* and *Arthrobacter,* as well as some strains related to the genera *Oceanobacillus*, *Halobacillus* and *Idiomarina.* In addition, many of the strains isolated from materials employed to stuff the mummies showed cellulolytic activities, such as those related to species of the genera *Chromohalobacter* and *Nesterenkonia*, as well as those identified as *Staphylococcus equorum* and *Halomonas* sp. Furthermore, many of the strains were pigmented ranging from yellow to a strong pink color, being directly related to the discoloration displayed by the materials.

## Introduction

Traditionally, halophilic microorganisms have been isolated from salted foods and hypersaline environments, mainly saline lakes, salterns and saline soils (Ventosa 2006). This group of microorganisms can be classified on the basis of their optimal NaCl requirements in different categories: slightly (optimal NaCl requirement of 1–3 %), moderately (optimal NaCl requirement of 3–15 %) and extremely halophilic which require more than 15 % of NaCl for optimal growth (Kushner 1978). Classically, two different groups of halophilic microorganisms have been identified in hypersaline environments: extremely halophilic archaea (haloarchaea) and moderately halophilic bacteria. Halophilic bacteria are currently represented by a large number of species included on different phylogenetic branches, reflecting their broad metabolic activities (Ventosa et al. 2012). In this study, no hypersaline lake or other extreme hypersaline environment was present, yet interestingly some wall and tow samples recovered in the Capuchin Catacombs of Palermo (Italy) were sufficient to isolate moderately halophilic bacteria.

**Fig. 1:**
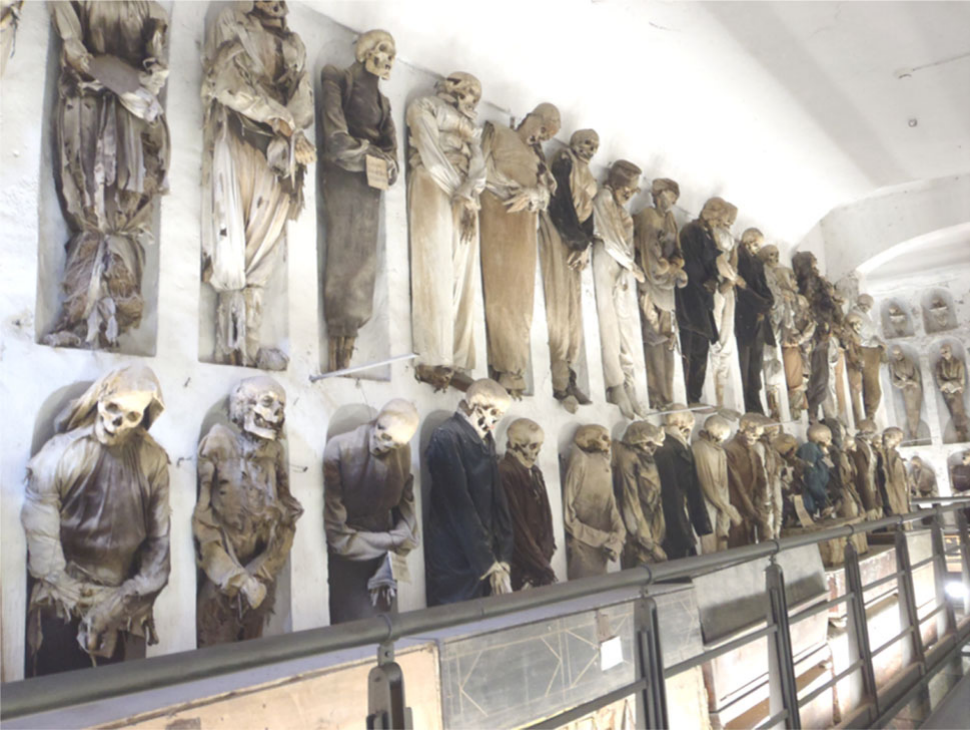
Mummies displayed along the walls of the Capuchin Catacombs of Palermo, Italy (pictures: Sterflinger)

**Fig. 2:**
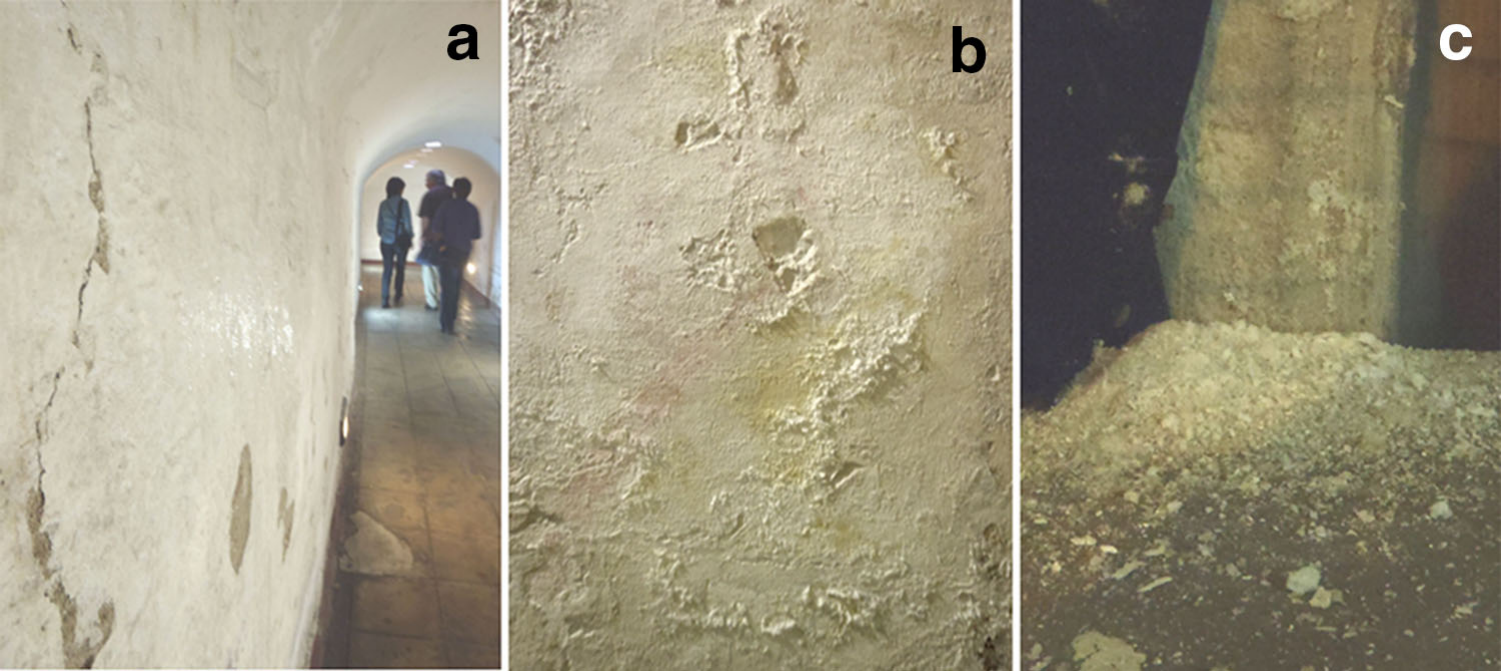
**a** Water migrating horizontally into the walls of the Catacombs. **b** Salt crusts of sodium chloride on the walls. **c** Detached salt contaminating other materials (pictures: Sterflinger)

The Catacombs of Palermo contain over 1800 preserved bodies dating from the 16th to 20th centuries AD, showing evidence of biodeterioration. Most of the mummies are positioned directly on the walls of the Catacombs (Fig. 1). Within the framework of the “Sicily Mummy Project”, a sampling campaign performed at the Catacombs in 2010 revealed an extensive rosy discoloration of the Catacombs’ walls, which were in direct contact with the surrounding soil. In some areas, water was migrating horizontally into the walls of the Catacombs (Fig. 2a), carrying with it soluble salts. Due to changes in physical parameters, salts from the solution precipitated at the exposed surface creating salt efflorescences (Amoroso and Fassina 1983), which appeared to be dispersed all over the walls (Fig. 2b). The crystallization of salts on the wall of the Catacombs has resulted in a destructive effect. Salts crystallized to different hydrates occupy a larger space and produce an additional pressure that has led to material loss and destruction due to cracking and detachment of the walls (Saiz-Jimenez and Laiz 2000). The detached material has accumulated on the surface of both coffins and mummies, producing further contamination of these artifacts (Fig. 2c). Moreover, the salt efflorescences deposited on the walls mimic the conditions found in extreme habitats, favoring the proliferation of halotolerant/halophilic microorganisms (Piñar et al. 2009). In a previous survey, an extensive microbiological and molecular investigation was performed at the Catacombs of Palermo (Piñar et al. 2013). Samples were taken from skin, muscle, hair, bone, stuffing materials, clothes and surrounding walls as well as from the air inside the Catacombs. The molecular techniques applied in that survey showed the dominance of halophilic species of the domains *Bacteria and Archaea* on the walls and, as a result of salt emanating from the walls, on some of the mummy-related materials. These microorganisms were thought to be responsible for the massive discoloration phenomena observed on the walls and stuffing materials. These results prompted further studies to focus on halophilic microorganisms that, in the past, were overlooked due to the use of unsuitable culture media with inadequate salt concentration, and/or the long incubation time required for the growth of this group of microorganisms under laboratory conditions (Piñar et al. 2001a).

**Fig. 3:**
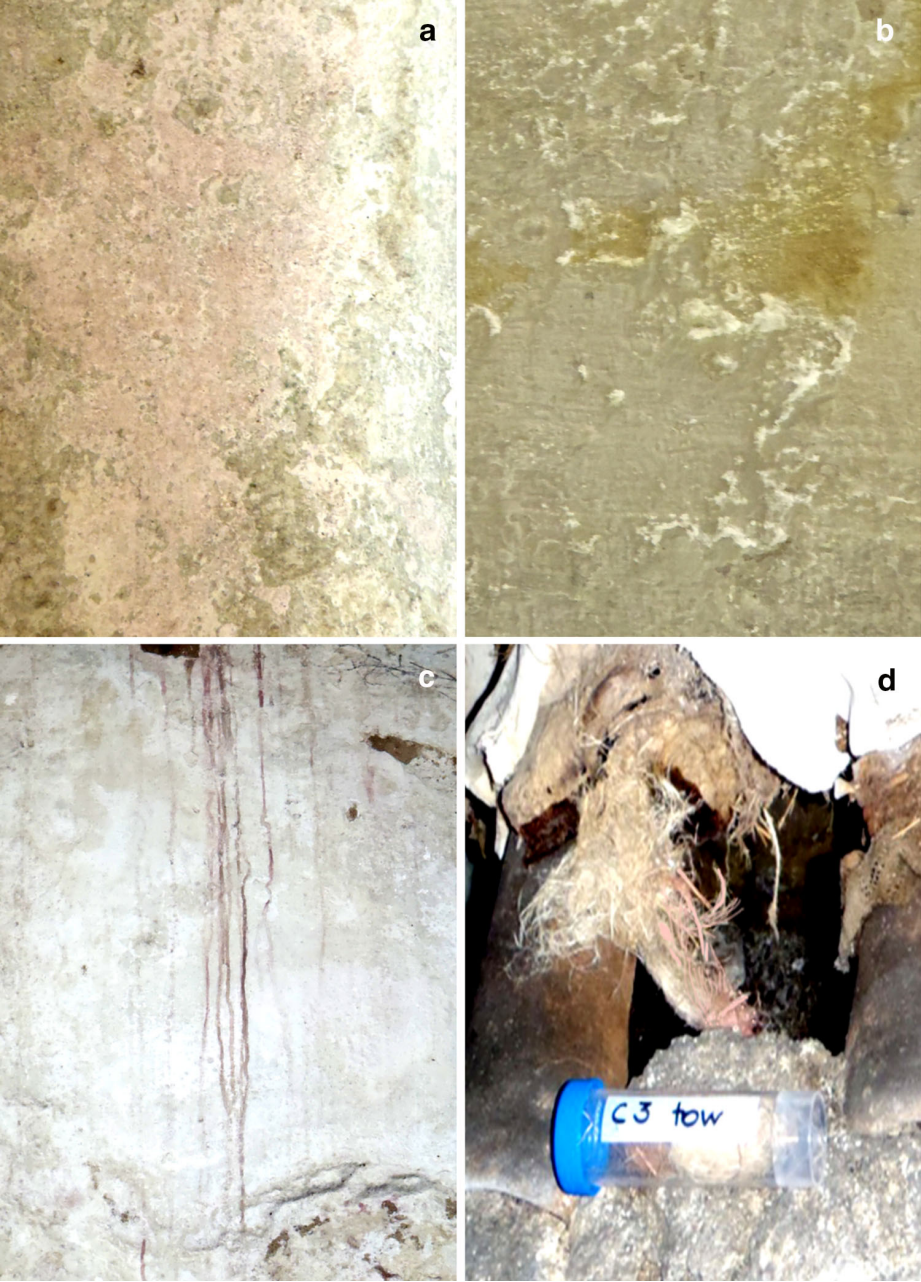
**a** Sample W1: rosy discolored wall. **b** Sample W2: salt efflorescence with yellowish discoloration. **c** Sample PS: wall showing purple stains. **d** Sample C3: stuffing material, showing orange to rosy discoloration (pictures: Sterflinger)

Several studies have been published focusing on the formation of pigmented biofilms and the discoloration of various stone materials. They have shown that salt-attacked monuments, wall paintings and artworks represent a common habitat for extremely salt-tolerant and moderately halophilic microorganisms (Ettenauer et al. 2010; Imperi et al. 2007; Ortega-Morales et al. 2004, 2005; Piñar et al. 2001a, b, c, 2009, 2013; Ripka et al. 2006; Rölleke et al. 1996, 1998; Saiz-Jimenez and Laiz 2000; Schabereiter-Gurtner et al. 2001). Microorganisms produce a wide variety of biogenic pigments such as chlorophyll, carotin, carotenoid, ruberin and melanin. The formation of orange to red pigments by halophilic microorganisms is due to the production of carotenoids. Carotenoids are isoprenoids containing a characteristic polyene chain of conjugated double bonds. The two general groups of pigments are the isoprenoid hydrocarbons (carotenes) and oxygenated derivatives (xanthophylls) (Köcher and Müller 2011). Carotenoids act to protect the cells against photooxidative damage (Köcher and Müller 2011; Oren 2009) as well as membrane stabilizers against chemical and/or salt stress (Köcher and Müller 2011). On salt-attacked objects, the inhabiting halophilic bacteria and haloarchaea usually form orange to pink or even violet stains (Fig. 3). The biogenic pigments are usually very stable on the materials even after the death of the causative microorganisms.

In this study, the cultivable fraction of the halophilic microflora inhabiting the walls and stuffing materials of the Catacombs of Palermo were investigated, to elucidate if they are involved in the discoloration phenomena observed on the different materials, and to clarify the potential biodegradative role of this peculiar microbiota in the further deterioration of the mummies and related materials. The objectives of this work were: (I) the isolation and molecular identification of the halophilic microbiota present on the walls and stuffing materials of the Catacombs, (II) the comparison of two different PCR-based methods for screening of the isolated strains, (III) to clarify the biological origin of the extensive rosy discoloration evidenced on the walls and other materials at the Catacombs and (IV) to test the biodegradative (proteolytic and cellulolytic) activities demonstrated by the isolated strains.

## Materials and methods

### Sampling

Three samples—W1: rosy discolored wall (Fig. 3a), W2: salt efflorescence with yellow discoloration (Fig. 3b) and PS: wall showing purple stains (Fig. 3c)—were collected and pooled from different areas of the walls of the Catacombs. In addition, sample C3, consisting of tow and straw used as stuffing material for the mummies and showing orange to rosy discoloration (Fig. 3d), was collected with sterile scalpels and forceps (Bayha GmbH, Germany).

### Cultivation strategy

Three different media were used for enrichment: Trypticase Soy Agar (TSA) supplemented with NaCl (3 %) and MgSO_4_·7H_2_O (2 %) (Laiz et al. 2009), Maintenance Medium (HMM, 10 % NaCl) (Spring et al. 1996) and M2 medium (20 % NaCl) (Tomlinson and Hochstein 1976). Enrichments were conducted in 300 ml Erlenmeyer flasks containing 30 ml medium. To avoid fungal growth, media were supplemented with 50 mg l^−1^ cycloheximide (Sigma). Flasks were incubated aerobically at 28 ^o^C by shaking at 180 rpm (Thermo Scientific Equipment MaxQ 8000, Germany). Over a total period of 4 weeks, weekly aliquots of 100 µl enrichments were serial-diluted and plated onto the same solid media. Plates were incubated aerobically at room temperature and at 28 ^o^C for 2–4 weeks, depending on the growth of the microorganisms. The cell morphology was examined on an Olympus SZX9 phase contrast microscope. Cells showing different morphology and appearance were transferred to new culture plates to obtain pure cultures. Pure isolates were cultivated in fresh media until exponential growth occurred, to be finally stored in 70 % glycerol at −80 ^o^C for preservation.

### Molecular screening of isolated microflora

Genomic DNAs were extracted according to the protocol provided by Ausubel et al. (1991). Bacterial strains were firstly selected by fluorescence ITS PCR (f-ITS) as described by Kraková et al. (2012). The internal transcribed spacer (ITS) between the 16S and 23S rRNA gene was amplified, and followed by separation of fluorescently labeled PCR products by capillary electrophoresis. Briefly, the PCR mixture contained 10 pmol of L1: CAAGGCATCCACCGT (Jensen et al. 1993) and G17: FAM-GTGAAGTCGTAACAAGG, FAM labeled primers (Kraková et al. 2012), 200 mmol l^−1^ dNTP, 1.5 U HotStarTaq plus DNA polymerase (Qiagen), 1x PCR buffer, and 6 µl of template DNA in the total reaction volume of 25 µl. The temperature program consisted of the initial denaturation at 95 ^o^C for 5 min, 35 cycles (95 ^o^C for 30 s, 54 ^o^C for 30 s, 72 ^o^C for 1 min) and a final polymerization at 72 ^o^C for 8 min. Resulting f-ITS products were separated on the automatic genetic analyzer ABI Prism 3100 Avant (Applied Biosystems, Foster City, CA, USA). DNA fragments were sized using the LIZ-600 DNA standard and GeneMapper 3.7 software (Applied Biosystems).

In addition, bacterial strains were subjected to Random Amplified Polymorphic DNA polymerase chain reaction (RAPD-PCR) analyses as described by Ripka et al. (2006). For RAPD analysis, the PCR was performed with three different primers (namely, primers D14216, D11344 and D14307), all of which were at least 17 nt in length (Ripka et al. 2006). Primer concentrations of 12.5 pmol ml^−1^ were applied to the 1x diluted Master Mix (Promega). The PCR was carried out in 25 and 2.5 µl of template was added. PCR reactions were executed in a MJ Research PTC-200 Peltier Thermal Cycler using PCR Master Mix (Promega, Mannheim, Germany). The cycling program was 4 cycles of [94 ^o^C, 5 min; 4 ^o^C, 5 min; and 72 ^o^C, 5 min; low stringency amplification], 30 cycles of [94 ^o^C, 1 min; 55 ^o^C, 1 min; and 72 ^o^C, 2 min; high stringency amplification] and a final elongation step for 10 min at 72 ^o^C (Welsh and McClelland 1990). The whole reaction batches were run with 4 µl loading dye solution (Fermentas) in a 2 % (w/v) agarose gel for ~130–160 min at 70 V, stained in an ethidium bromide solution (1 mg/ml; stock 10 mg/ml) for 30–45 min and visualized by a UVP documentation system (BioRad Transilluminator, Universal Hood; Mitsubishi P93D-printer). The GeneRulerTM 100 bp DNA ladder from Fermentas was used as a size marker.

### 16S rDNA sequencing and phylogenetic analyses

For sequencing analyses of bacterial isolates, ~1200 base pair 16S rDNA fragments were amplified using the forward primer 27f and the reverse primer 1492r (Lane 1991). 100 µl PCR reaction volumes separated into two tubes to 50 and 3 µl of the extracted DNA were conducted. The thermocycling program used was as follows: 5 min denaturation at 95 ^o^C, followed by 30 cycles consisting of 1 min denaturation at 95 ^o^C, 1 min primer annealing at 55 ^o^C and 2 min primer extension at 72 ^o^C, followed by a final extension step of 5 min at 72 ^o^C. The products obtained were purified using the QIAquick PCR Purification Kit (Qiagen) and analyzed by electrophoresis in 2 % (w/v) agarose gels. PCR products were externally sequenced by Sanger sequencing with a fleet of 16 ABI 3730xl (GATC Biotech, Germany). Comparative sequence analyses were performed by comparing pairwise insert sequences with those available in the online databases provided by the National Centre for Biotechnology Information using the BLAST search program (Altschul et al. 1997). The ribosomal sequences of the bacterial strains have been deposited in the EMBL nucleotide database under the accession numbers listed in Table 1.

**Table 1:** Grouping according to f-ITS and RAPD-PCR, phenotypic characteristics, phylogenetic classification and biodegradative tests of the isolated bacterial strains

[NaCl] (%)	f-ITS profiles (bp)	Strains grouped by RAPD	Phenotype	Closest phylogenetic relative	Similarity (%)	Accession Nr.	Proteolytic test	Cellulolytic test
**Sample W1—rosy discolored wall**								
3	I_3_: (670, 760)	*W1*-*1a*, W1-1b, W1-3, W1-4, W1-5, W1-6	Salmon–yellowish colonies	*Idiomarina* sp. [KC753341, JF521499, AB167046]	99.0	KJ145754	+	n.d.
10	I_10_: (261, 283, 293, 308, 402, 405, 457, 463)	*W1*-*7*, W1-8	White, bright colonies	*Oceanobacillus oncorhynchi* [JN409467, NR_042257]	99.0	KJ145755	−	n.d.
	II_10_: (365, 449, 633)	*W1*-*9*, W1-14	Salmon, bright colonies	*Virgibacillus* sp. [EU277749, KC013356]	98.0	KJ145756	++	n.d.
	III_10_: (600, 651, 657, 709, 726)	*W1*-*10*, W1-12	Salmon–yellowish colonies	*Idiomarina* sp. [JF521499, AB526349, FJ746576]	99.0	KJ145757	w.g.	n.d.
	IV_10_: (597, 654, 656, 725)	*W1*-*11*	Beige-salmon colonies	*Virgibacillus picturae* [AJ276808]	99.0	KJ145758	+	n.d.
	V_10_: (365, 590, 629, 637, 808)	*W1*-*13*	Yellow, bright colonies	*Oceanobacillus* sp. [FJ424491, NR_028952]	100	KJ145759	+	n.d.
20	I_20_: (387, 411)	*W1*-*15*	Beige colonies	*Halobacillus virgiliensis* [AM161501, AM161499]	99.0	KJ145760	–	n.d.
		*W1*-*16*	Pink colonies	*Halobacillus virgiliensis* [AM161501, AM161499]	99.0	KJ145761	–	n.d.
**Sample W2—wall showing salt efflorescence with yellow discoloration**								
3	II_3_: (310, 343, 431, 435)	*W2*-*1a*, W2-1b, W2-3, W2-4a, W2-4b	Beige big colonies	*Bacillus aryabhattai* [KF208483]	99.0	KJ145762	+	n.d.
	III_3_: (232)	*W2*-*2*	Beige, small colonies	*Lysinibacillus* sp. [KF208480, KF788075]	99.0	KJ145763	–	n.d.
10	VI_10_: (280, 299, 301, 340, 526, 543)	*W2*-*5*	Beige–yellowish colonies	*Halobacillus hunanensis* [FJ425898] *Halobacillus naozhouensis*, [HG515391]	99.0	KJ145764	+	n.d.
	VII_10_: (480, 649, 689, 811)	*W2*-*6*, W2-7	Salmon–yellowish, bright colonies	*Idiomarina* sp. [KC753341, HE586864, JF521499]	99.0	KJ145765	w.g.	n.d.
20	II_20_: (386, 411)	*W2*-*8*, W2-9, W2-10, W2-11	Yellow–orange colonies	*Marinococcus* sp. [HG515394, AY987847]	99.0	KJ145766	–	n.d.
**Sample PS—wall showing purple stains**								
3	IV_3_: (215, 232, 457)	*PS1a*, PS1b, PS3a, PS3b, PS5	Beige colonies	*Bacillus* sp. [JN230359, JN717163, JN082261]	99.0	KJ145767	+++	n.d.
	V_3_: (636, 680, 764)	*PS4*, PS6	Yellow, bright colonies	*Arthrobacter protophormiae* [NR_026195, JX007987, EU086815]	99.0	KJ145768	+++	n.d.
10	VIII_10_: (628, 669, 761)	*PS7*	Salmon–orange, bright colonies	*Halomonas* sp. [HM587244, EU308349]	98.0	KJ145769	–	n.d.
	IX_10_: (694, 796)	*PS7b*	Salmon–yellowish, bright colonies	*Oceanobacillus picturae* [AB489113, EF512727, EF512732]	99.0	KJ145770	+	n.d.
	X_10_: (662, 669, 742, 754, 811)	*PS8*	Beige, big colonies	*Bacillus* sp. [FJ373031, AJ315068, Q030919]	99.0	KJ145771	++	n.d.
	XI_10_: (651, 699, 730)	*PS9*	Beige colonies	*Halomonas* sp. [KF682368, KC884689, JQ716247]	99.0	KJ145772	w.g.	n.d.
	XII_10_: (689, 693, 794, 812)	*PS10*, PS11	Salmon–orange colonies	*Bacillus* sp. [HQ699497, JQ716212, JF411285]	99.0	KJ145773	+++	n.d.
20	III_20_: (331, 383, 386, 411, 421, 453, 483, 597)	PS12, *PS14*,	Beige, small colonies	*Oceanobacillus iheyensis* [BA000028]	99.0	KJ145774	–	n.d.
	IV_20_: (295, 319, 357, 547, 562)	*PS16*	Beige colonies	*Halobacillus styriensis* [AM161506, AM161507]	99.0	KJ145775	+	n.d.
	VI_10_: (280, 299, 301, 340, 526, 543)	PS13, PS15, PS17, *PS18*, PS19a	Dark pink colonies	*Halobacillus hunanensis [FJ425898]* *Halobacillus naozhouensis, [HG515391]*	99.0 99.0	KJ145776	+	n.d.
		*PS20*	Dark pink colonies	*Halobacillus hunanensis [FJ425898]* *Halobacillus naozhouensis, [HG515391]*	99.0	KJ145777	+	n.d
		*PS21*	Beige–yellowish colonies	*Halobacillus hunanensis [FJ425898]* *Halobacillus naozhouensis, [HG515391]*	99.0	KJ145778	+	n.d.
		*PS22*, PS23	Salmon colonies	*Halobacillus* sp. [AB695093]	99.0	KJ145779	–	n.d.
		*PS24*	Dark pink colonies	*Halobacillus hunanensis [FJ425898]* *Halobacillus naozhouensis, [HG515391]*	99.0	KJ145780	+	n.d
	V_20_: (347, 367, 371, 377, 399, 450, 456, 557, 564)	*PS19b*	Beige, small colonies	*Staphylococcus equorum* [KF439737, KF439739, KC844770]	99.0	KJ145781	–	n.d.
**Sample C3—stuffing material showing orange to rosy discoloration**								
3	VI_3_: (246, 266, 299, 455, 466)	*C3*-*1a*, C3-1b	Beige, big mucous colonies	*Bacillus endophyticus* [KF254667, HQ844498, HM753612]	99.0	KJ145782	+	–
	VII_3_: (636, 691, 783)	*C3*-*2*	Yellow, bright colonies	*Halomonas* sp. [KF682368, KC884689, JQ716248]	99.0	KJ145783	–	–
	VIII_3_: (266, 592, 668, 755)	*C3*-*3*	Salmon, bright colonies	*Halomonas* sp. [EF512731, EF512710]	99.0	KJ145784	–	–
	IX_3_: (602, 657, 725)	*C3*-*4*, C3-5	White, bright colonies	*Staphylococcus* sp. [FN994187, FJ386519]	99.0	KJ145785	–	–
10	XIII_10_: (693, 730, 797)	*C3*-*6*, C3-11	Beige, big colonies	*Staphylococcus equorum* [KF439737, KF439739, KC844770]	99.0	KJ145786	–	+
	XIV_10_: (689, 692, 795)	*C3*-*7*, C3-8	Beige, small colonies	*Chromohalobacter nigrandesensis* [NR_042011]	99.0	KJ145787	–	+
	XV_10_: (420, 454, 534, 550)	*C3*-*9*	Beige–salmon colonies	*Halomonas* sp. [EU440966, DQ659441]	99.0	KJ145788	–	–
	XVI_10_: (406, 454, 551)	*C3*-*10*	Yellow colonies	*Halomonas* sp. [EU088257, FN257742, JQ716247]	98.0	KJ145789	–	+
	XVII_10_: (274, 302, 381, 400, 418, 671)	*C3*-*12*, C3-14, C3-15	Intense orange colonies	Uncultured bacterium microbiome [HM292641] *Brevibacterium permese* [NR_025732]	98.0 97.0	KJ145790	–	-/+
	XVIII_10_: (386, 411)	*C3*-*13*	Yellow colonies	*Virgibacillus halodenitrificans* [NR_042967, AB697708, AB697710]	99.0	KJ145791	+	–
20	VI_20_: (250, 781)	*C3*-*16*, C3-17, C3-18, C3-19	Beige, small colonies	*Chromohalobacter nigrandesensis* [NR_042011]	99.0	KJ145792	–	+
	VII_20_: (247, 298, 339, 412, 526)	*C3*-*20*, C3-21, C3-22, C3-23	Beige, big colonies	*Nesterenkonia halobia* [NR_026197] *Nesterenkonia halophila* [NR_043205]	99.0 99.0	KJ145793	–	+

*n.d.* not determined, *w.g*. weak growth, *+* positive reaction, *++* strong positive reaction, *+++* very strong positive reaction

f-ITS profiles: I_3_–IX_3_; I_10_–XVIII_10_; I_20_–VII_20_, the size, in bp, of the amplified fragments is shown in brackets

Strains marked in italics were subjected to sequencing and phylogenetic analyses

### Biodegradative plate assays

The proteolytic activity of the isolated microflora was tested by cultivation on gelatin agar plates (R2A-Gel) with different NaCl percentages (3, 10 and 20 %). The gelatin medium was prepared using R2A agar (Oxoid, Basingstoke, UK) plus 3 or 10 % of NaCl. This medium was autoclaved separately and then 0.4 % of sterilized gelatin was added (Sigma-Aldrich, Germany) as well as an amount of a solution of 20 % MgSO_4_ autoclaved separately to reach into the agar with a final MgSO_4_ concentration of 2 %. The R2A agar with 20 % of NaCl was prepared as the above media, but rather than the MgSO_4_ solution, 50 ml of another solution, sterilized separately and composed by 40 % of MgCl_2_ × 7 H_2_O, 4 % of KCl and 0.4 % of CaCl_2_ × 2 H_2_0, were added into 950 ml of agar medium. After the growth of the microorganisms, to have a better visualization of the hydrolysis zone, a 10 % tannin solution was flooded on the agar plates (Saran et al. 2007). All proteolytic assays were performed in triplicate using 60-mm plates incubated at room temperature generally for 5–7 days.

The cellulolytic ability was checked using R2A agar with different salt concentrations (3, 10 and 20 %) and prepared following the same procedures described above. Then the media were supplemented with 0.2 % hydroxyethylcellulose containing 13.5 % (w/w) of covalently linked Ostazin Brilliant Red H-3B (OBR-HEC, Institute of Chemistry, Slovak Academy of Sciences, Bratislava, Slovakia), which was autoclaved separately. On one agar plate, it was possible to test six strains at the same time, and a clear zone appeared around the cellulolytic microorganisms. The test was performed in triplicate and the plates were incubated at room temperature generally from 5 to 7 days.

## Results

### Isolation of bacterial strains

A total of 80 isolates differing in morphology and appearance could be cultivated from the four collected samples—16 strains (20 %) from sample W1, 13 strains from sample W2 (16.25 %), 27 strains from sample PS (33.75 %), and 24 strains from the stuffing material sample C3 (30 %). It is important to note that many of the isolated strains, especially those isolated from media containing higher NaCl concentrations, displayed a strong pigmentation ranging from yellow–orange to dark pink (Table 1).

Summarized over all 80 cultured strains, 25 isolates were derived from 3 % NaCl supplemented media (31.25 %), 27 strains from 10 % NaCl media (33.75 %), and 28 strains from 20 % NaCl media (35 %). To screen the bacterial isolates for sequencing and further characterization, a genotyping strategy was applied.

### Bacterial selection and clustering

**Fig. 4:**
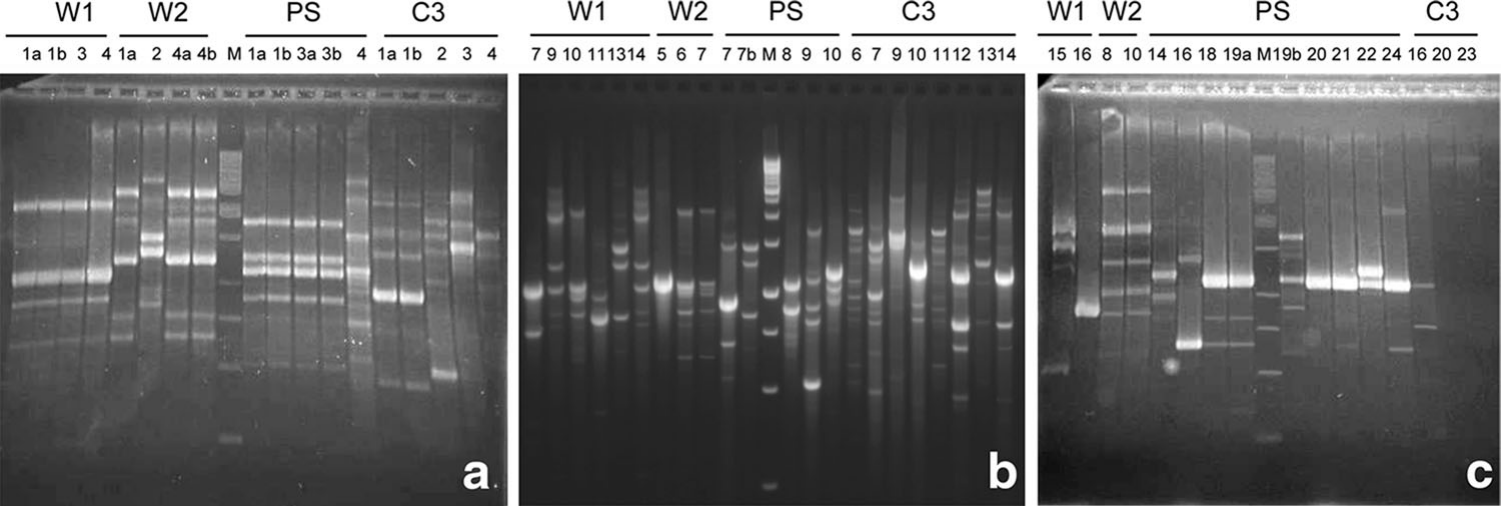
RAPD-PCR patterns of representative strains isolated from **a** 3 % NaCl, **b** 10 % NaCl, and **c** 20 % NaCl media. The number of lanes indicates the number of the strains. *Lanes M* 1 kb ladder (Fermentas)

Two PCR-based techniques were used for the genotyping of the isolated strains. The f-ITS method is based on the amplification of the internal transcribed sequences between the bacterial 16S and 23S rDNA. RAPD-PCR uses arbitrarily chosen primers to prime DNA synthesis from pairs of sites with partial or complete matching and, as a result, DNA profiles are obtained based on variations in the entire bacterial genome. In this study, RAPD analyses were performed with three different primers as mentioned in the Methods section. Among the three tested primers, the 23 nt primer D11344 proved to be the most discriminative (Fig. 4), originating RAPD-PCR profiles with higher band numbers. However, when using the other two primers (D14307 and D14216), not all isolates could be identified by type (data not shown).

Results derived from both PCR-based typing techniques, f-ITS and RAPD (using primer D11344), yielded the same conclusions, allowing the selection of 9 different fingerprints among the strains isolated from the 3 % NaCl medium, 5 of which came from wall samples and 4 from stuffing materials (Fig. 4a). The same was observed for the strains isolated from 10 % NaCl. Both f-ITS and RAPD analyses allowed the identification of 18 different profiles, 12 of which were from wall samples and 6 were from stuffing materials, respectively (Fig. 4b). When the strains isolated from 20 % NaCl media were analyzed by both fingerprints PCR techniques, a certain discrepancy was evident: in fact, 12 and 7 different profiles were, respectively, produced by RAPD (Fig. 4c) and f-ITS. The main difference concerned the RAPD clusters including the isolates PS13, PS15, PS17, PS18, PS19a (cluster 1); PS20 (cluster 2), PS21 (cluster 3); PS22, PS23 (cluster 4); PS24 (cluster 5), which were grouped by f-ITS in a unique cluster (VI_10_). These resulted in the same cluster of the strain W2-5 isolated on the 10 % NaCl medium (see Table 1). The strains W1-15 and W1-16, differentiated by RAPD, were also included in the same f-ITS cluster (I_20_). Finally, the strains C3-20 and C3-23, non-typeable by RAPD, produced the same f-ITS profile (VII_20_) together with the isolates C3-21 and C3-22.

One interesting point was the case of different *Staphylococcus* strains, i.e., *S. equorum* (strains PS19b, C3-4, C3-5, and C3-6, C3-11) isolated from different media, which were separated by RAPD and f-ITS (V_20_, IX_3_, and XIII_10_) in three different clusters corresponding to diverse salt concentrations. The data regarding the clustering of all isolated strains are showed in Table 1.

### Phylogenetic identification of selected bacterial strains

One representative strain of each RAPD cluster was selected for identification by 16S rDNA sequencing. The isolate C3-20, not typeable by RAPD analyses (see Fig. 4c), was also selected for identification. The phylogenetic identification of the selected strains obtained by comparison of sequences derived from the isolated strains with sequences of known bacteria in the EMBL database using the search tools FASTA and BLAST is summarized in Table 1.

Similarity values with sequences from the EMBL database ranged from 98 to 100 %. Most of the isolates were affiliated with cultured bacterial strains, but the strain C3-12 was mostly related to an uncultured cloned sequence from the microbiome, and with less similarity (97 %) with cultured species of the genus *Brevibacterium*. Results from 16S rDNA sequence analysis revealed that all 80 strains were affiliated with species of 13 genera within three phyla: *Firmicutes*, of the order Bacillales (number of isolates, *n* = 49, representing 61.25 % of all isolated strains), *Proteobacteria*, of the *Gamma*-class (*n* = 22, 27.5 %), and *Actinobacteria* (*n* = 9, 11.25 %).

Within the *Firmicutes* phylum, a total of seven different genera were identified, with the genera *Bacillus* and *Halobacillus* being the two most abundant (representing 18.75 and 17.5 % of all isolated strains, respectively). The *Oceanobacillus* genus accounted for 7.5 % of the total strains and the *Staphylococcus* genus for 6.25 %. In addition, the genera *Virgibacillus* and *Marinococcus* accounted for 5 % each of the total strains, while finally, the genus *Lysinibacillus* accounted for 1.25 % of the total strains. Within the *Gammaproteobacteria*, three different genera were identified, namely the genera *Idiomarina* (12.5 % of total strains), *Halomonas* and *Chromohalobacter* (each 7.5 %). Finally, three genera showed to be affiliated with the phylum *Actinobacteria*, namely the genus *Nesterenkonia* (5 % of strains), uncultured strains most related to the genus *Brevibacterium* (3.75 %) and the genus *Arthrobacter* (2.5 %).

### Biodegradative characterization

The 80 isolated bacterial strains were screened for their proteolytic activity and, in addition, those strains isolated from stuffing materials were tested for their cellulolytic properties.

Many of the bacterial strains isolated from wall materials displayed proteolytic activities. All strains related to species of the genera *Bacillus* and *Virgibacillus*, also isolated from stuffing materials, showed a strong proteolytic activity (Table 1). The genus *Oceanobacillus* showed a different behavior depending on the species detected. Those strains most related to *O.* *oncorhynchi* and *O.* *iheyensis* showed negative results and those related to *Oceanobacillus* sp. and *O. picturae* showed positive results in the proteolytic test. The same was observed for the genus *Halobacillus*. Strains most related to *H.* *virgiliensis* showed no proteolytic activities and those most related to *H. styriensis* and *H. hunanensis* showed positive results. The rest of the strains related to members of the *Firmicutes*, affiliating to species of the genera *Lysinibacillus*, *Marinococcus* and *Staphylococcus,* showed no proteolytic activities (Table 1).

Within the *Gammaproteobacteria*, strains related to *Idiomarina* sp., those isolated from wall samples W1 and W2, displayed proteolytic activities only when the bacteria were grown in the 3 % NaCl medium, but not in the 10 % NaCl medium. However, the reason behind this could be the very weak growth of these strains in the latter culture medium (Table 1). Strains related to species of the genera *Halomonas* and *Chromohalobacter*, mainly isolated from stuffing materials, showed no proteolytic activities.

Within the *Actinobacteria*, strains related to *Arthrobacter protophormiae*, isolated only from wall sample PS, showed a strong proteolytic activity. In contrast, strains mostly related to uncultured clones from the microbiome and to *Nesterenkonia halobia,* only isolated from stuffing materials, showed negative results in the proteolytic test.

The cellulose-rich content of the materials used as stuffing for the mummies, tow and straw, prompted the further cellulolytic test on the strains isolated from these materials. Strains related to *Chromohalobacter* sp. (*C.* *nigrandesensis*) and to *Nesterenkonia* sp. (*N. halobia* and *N. halophila*) all showed cellulolytic activities. Strains related to *Staphylococcus* sp. and to *Halomonas* sp. showed a variation depending on the strains, some of them being positive and some of them negative in the cellulolytic test (Table 1). The same was observed for the strains related to an uncultured clone from the microbiome.

## Discussion

### Cultivation analyses

It is well known that the use of standard cultivation techniques enables only a small proportion of the total inhabiting bacterial population to be cultivated (Amann et al. 1995; Ward et al. 1990). When dealing with the isolation of halophilic microorganisms, the preparation and use of hypersaline media reveal special challenges unique to high salinities, as well as the same concerns regarding any microbial culture system (Schneegurt 2012). Therefore, three different culture media that were previously used with success for the isolation of moderately halophilic microorganisms were applied in this study (Laiz et al. 2009; Spring et al. 1996; Tomlinson and Hochstein 1976). As a result, 80 halophilic bacteria were isolated from three wall samples showing orange to rosy and even purple discoloration as well as salt efflorescences and from the tow and straw used as the stuffing material for the mummies displayed in the Catacombs. Moderately halophilic bacteria grow optimally in media containing 3–15 % (w/v) salt, but they can also grow at as high concentrations as 25 % (Ventosa et al. 1998). This is the case of the species of *Idiomarina*, *Halomonas*, *Oceanobacillus*, *Virgibacillus, Marinococcus* and *Chromohalobacter* (Gurtner et al. 2000; Heyrman and Swings 2001; Heyrman et al. 2002, 2003; Ivanova et al. 2000; Lu et al. 2001; Maturrano et al. 2006; Peng et al. 2009; Prado et al. 2006; Ripka et al. 2006; Romano et al. 2006; Wang et al. 2008; Yoon et al. 2004) and some species of the genera *Bacillus* and *Staphylococcus* (El-Rahman et al. 2002; Heyrman and Swings 2001; Jeong et al. 2013) that showed to be the closest relatives of the strains isolated in this study. Interestingly, the *Nesterenkonia* species were only isolated in media containing 20 % NaCl, in spite of the fact that they are considered to be moderately halophilic (Li et al. 2008). A similar behavior was observed for the species of the *Halobacillus* genus, which were mainly isolated in media containing 20 % NaCl and could be isolated in the medium containing 10 % NaCl only in the case of sample W2.

### Comparison of the two PCR-based typing methods for screening of the isolated strains

The two PCR-based molecular methods used for screening the isolated bacteria, f-ITS and RAPD, allowed differentiation between the 80 isolated strains in an early selection step. This previous screening facilitates microbiologists to choose among several microorganisms possessing similar morphology after the isolation step. The f-ITS-PCR has already been tested for the screening of microorganisms in different environments (Bućková et al. 2013; Kraková et al. 2012; Pangallo et al. 2008; 2009), and its usefulness in selecting isolated bacteria was reconfirmed in this study. However, when comparing f-ITS-PCR for genotyping of bacterial strains with RAPD analysis, it turned out that the latest PCR-based method possessed a higher discriminatory power. The discrepancy was evident mainly for several *Halobacillus hunanensis* strains which were included in only one cluster (VI_10_) by f-ITS, while RAPD analyses divided them among 5 different groups. This can be due to the fact that the f-ITS-PCR is only able to discriminate genetic variations occurring within the internal transcribed sequences between the bacterial 16S and 23S rDNA analyzed. In contrast, RAPD-PCR uses an oligonucleotide of an arbitrarily chosen sequence to prime DNA synthesis from pairs of sites with which it partially or completely matches. The DNA profiles obtained allow discrimination at the subspecies level based on the DNA diversity in the entire bacterial genome, offering a broad spectrum of genetic variation (Welsh and McClelland 1990; Williams et al. 1990). This study further supports the fact that RAPD-PCR profiling reveals more detailed results and sub-classifies more strains at the species level (Ettenauer et al. 2010; Ripka et al. 2006).

Nevertheless, it is worth noting that results derived from RAPD-PCR analyses are strongly influenced by the primer used. In this study, RAPD profiles were obtained and compared by the independent use of three different primers, as mentioned in the Methods section. The three primers were at least 17 nt and contained 44–55 % GC. The differences in their GC content may affect annealing behavior (Power 1996). Primer D11344, at a concentration of 12.5 pmol per PCR, showed the most informative RAPD-PCR profiles (higher band numbers), confirming the results of previous studies (Ettenauer et al. 2010; Ripka et al. 2006). When using the other primers (D14307 and D14216) not all isolates proved to be typeable.

Those strains isolated at different NaCl concentrations and showing different RAPD profiles were subjected to sequencing and the results obtained were compared with sequencing results previously obtained from the same samples, when molecular analyses were applied to elucidate their biodiversity (Piñar et al. 2013).

### Comparison of the cultivable and non-cultivable fraction of the microbiota present on wall samples and stuffing materials

As previously reported in other studies (Donachie et al. 2007; Laiz et al. 2003; López-Miras et al. 2013a, b), culture-dependent and -independent methods yielded different results from the same sample when the results obtained in this study and those from our previous molecular survey at the Catacombs (Piñar et al. 2013) were compared. The order Bacillales of the phylum *Firmicutes* proved to be dominant on the enrichment cultures of the three wall samples, representing 50, 84.6 and 85.2 % of the strains isolated from samples W1, W2 and PS, respectively. On the stuffing material (sample C3), it was represented by 29.2 % of the isolated strains. Nevertheless, members of this order were not detected by molecular techniques on sample PS and C3, but they were on samples W1 and W2, and with a lower proportion in the clone libraries (21 and 28.3 % of clones for W1 and W2, respectively) (Piñar et al. 2013). Members of the phylum *Proteobacteria* (of the *Gamma*-class) were isolated from all wall samples as well as from the stuffing material, contributing 50, 15.4 and 7.4 % of the strains isolated from wall samples W1, W2 and PS, respectively, and 41.6 % of the strains isolated from sample C3. However, the cultivated fraction of the *Gammaproteobacteria* differed from that detected in the clone libraries of these samples (39.5, 34.8 and 13.6 % of clones for W1, W2 and PS, respectively, and 51.2 % for sample C3) (Piñar et al. 2013). Members of the phylum *Actinobacteria* were recovered from the enrichment cultures of samples PS and C3 (7.4 and 29.2 % of the strains isolated from sample PS and C3, respectively), but not from the enrichment cultures of samples W1 and W2. Nevertheless, actinobacteria were represented by sequenced clones in the clone libraries of all four samples investigated (21, 32.6 and 27.3 % for samples W1, W2 and PS, respectively, and 43.9 % for sample C3) (Piñar et al. 2013). The previous molecular survey also revealed the presence of sequences related to members of the phylum *Bacteroidetes* in all three wall samples that could not be isolated from the enrichment cultures. Attempts to isolate members of the phylum *Bacteroidetes* from salt-attacked monuments, which were previously detected by molecular methods, also showed to be unsuccessful in previous studies (Ettenauer et al. 2010).

Sample PS showed the highest level of bacterial diversity when molecular methods were applied, and in addition, sequences related to members belonging to the *Acidobacteria*, *Chloroflexi*, *Gemmatimonadetes* and *Nitrospirae* phyla were also detected (Piñar et al. 2013). None were retrieved as was expected, when using 3–20 % NaCl in the enrichment cultures conducted with this sample.

Furthermore, molecular analyses enabled the detection of the domain *Archaea* on salt efflorescences in sample W2, with sequences related to uncultured archaeons and to members of the genera *Halococcus* and *Halobacterium.* However, *Archaea* could not be isolated from the enrichment cultures of sample W2. The unsuccessful cultivation of these halophilic microorganisms on laboratory media, even using appropriate NaCl concentrations (20 % NaCl), was already reported (Piñar et al. 2009; Ettenauer et al. 2010).

It is worth noting that the genera identified by both approaches did not always coincide. On sample W1, halotolerant and halophilic species related to the genera *Salinisphaera* and *Halomonas* (*Gammaproteobacteria*) were detected by molecular techniques. However, they could not be isolated by cultivation techniques while *Idiomarina* sp. could. The same was observed for the *Firmicutes* of the order Bacillales. Sequences related to *Paenibacillus* sp. were detected by molecular means, but could not be isolated. In contrast, species related to the genera *Oceanobacillus*, *Virgibacillus* and *Halobacillus*, being dominant on the enrichment culture, could not be detected by molecular means.

On sample W2, halotolerant and halophilic species related to the genera *Salinisphaera*, *Halomonas* and *Idiomarina* were detected by molecular techniques, since it was possible to isolate only species belonging to the latter genus. The phylum *Firmicutes* was represented by species of the genus *Sediminibacillus*, *Bacillus* and *Thermoactinomyces* when analyzed by molecular methods. Species of the genera *Bacillus*, *Lysinibacillus*, *Halobacillus* and *Marinococcus* were isolated by culture-dependent approaches, but only species of *Bacillus* were detected by both approaches.

On sample PS, molecular analyses showed that the *Gammaproteobacteria* were represented by sequences related to the genera *Cellvibrio* and *Moraxella*, as well as by uncultured clones (Piñar et al. 2013). No representatives of these genera were retrieved from the enrichment culture of this sample, but instead species of the genus *Halomonas* were. Within the *Actinobacteria,* sequences related to the genera *Rubrobacter*, *Nocardioides*, *Mycobacterium* and *Pseudonocardia* were detected by molecular methods, but in contrast, species of the genus *Arthrobacter* were isolated. In addition, species of the genera *Staphylococcus*, *Bacillus*, *Oceanobacillus*, and *Halobacillus*, belonging to the phylum *Firmicutes*, were isolated from this sample, but its culture-independent analysis did not yield any clones harboring these sequences. A similar pitfall of molecular analysis was already observed (Ettenauer et al. 2010; Piñar et al. 2009) investigating the microflora colonizing salt-attacked monuments and has also been discussed by several authors (Laiz et al. 2003; López-Miras et al. 2013a, b).

In general, species of the genera *Bacillus* and *Halobacillus* were the most commonly isolated strains from wall samples. Strains belonging to species of these two genera were also the most commonly isolated strains in other studies attempting the isolation of halophilic microorganisms from salt-attacked monuments suffering from rosy biofilms (Ettenauer et al. 2010; Piñar et al. 2001a; Ripka et al. 2006). The viability of these organisms in such extreme habitats may be due to their osmotic adaptation through the synthesis of compatible solutes, such as glutamate, proline, ectoine and hydroxyectoine (Pittelkow and Bremer 2011) and their ability to survive unfavorable conditions as spores (Nicholson 2002), being able to regrow when favorable conditions are available.

On sample C3, removed from the stuffing material, sequences related to halotolerant and halophilic species of the genera *Salinisphaera* and *Chromohalobacter* were detected by molecular means (Piñar et al. 2013), as it was possible to isolate species of the latter genus from the enrichment culture, in addition to *Halomonas* sp. The *Actinobacteria* detected by molecular-based methods on this material proved to be affiliated with species of the genera *Arthrobacter*, *Brachybacterium*, *Cellulomonas* and *Kocuria*, as well as with uncultured bacterial clones associated with the microbiome (Kong et al. 2012). Using culture-dependent methods, it was also possible to isolate strains most related to uncultured bacterial clones associated with the microbiome (Kong et al. 2012), as well as species of *Nesterenkonia*, not detected by molecular methods. Species of *Nesterenkonia* were also isolated from other salt-attacked monuments (Ettenauer et al. 2010). In addition, species of the genera *Staphylococcus*, *Bacillus* and *Virgibacillus*, belonging to the phylum *Firmicutes*, were isolated from this sample but no clones harboring these sequences were detected in its clone library (Piñar et al. 2013).

Summarizing, the disparities in the results obtained by culture-dependent and—independent techniques in this study again demonstrate the drawbacks of each approach for an accurate description of the microbial community in a certain habitat. While giving a more complete picture of the microbial diversity of the studied environment, culture-independent approaches mainly based on rRNA gene detection are not supplying any information about the biodegradative potential of the detected cloned sequences. In contrast, classical cultivation techniques are incapable of obtaining all members of a microbial community, but only those which are able to grow on the supplied culture media (Giovannoni et al. 1990; Hugenholtz et al. 1998; Ward et al. 1990). However, they offer the possibility to isolate the microorganisms, and to investigate their biodeteriorative capabilities in relation to a given phenomenon. Moreover, in this particular study, cultivation allowed the ability to visualize the pigmentation of the grown strains as an important agent of the aesthetical damage displayed by the investigated objects and to characterize the biodegradative capabilities of the isolated strains.

### Biodegradative activities of the isolated strains

Many of the bacterial strains isolated from the wall materials displayed proteolytic activities. All isolated strains related to species of the genera *Bacillus*, *Virgibacillus* and *Arthrobacter* showed a strong proteolytic activity (Table 1), and so did the strains belonging to some species of the genera *Oceanobacillus*, *Halobacillus* and *Idiomarina*. These results are complementing those obtained in our previous survey at the Catacombs—through a culture-independent approach—in which, besides the halophilic microbiota, specialized microorganisms with potential degradative activities were present on and inside the mummy materials (Piñar et al. 2013). The halophilic microbiota was considered as mere contamination—due to salt detachment—and was thought to be responsible only for the rosy discoloration. Now, we know that this microbiota also possesses biodegradative abilities (proteolytic activities) that produce the degradation of the mummies positioned in direct contact with the walls (Fig. 1). The exchange of microbiota between the walls and the mummies was already notified on our previous survey, in which the presence of sequences related to pathogenic microorganisms and the human skin microbiome was detected on wall samples. In addition, some typical bacteria from the walls were detected on the bodies. The finding of these sequences was attributed to the fact that the remains were positioned directly on the walls, allowing a direct contact between wall and bodies and associated materials and the cross-contamination of these materials by microorganisms (Piñar et al. 2013).

In contrast, the cellulolytic activities detected on the strains isolated from the cellulose-rich materials used for stuffing the mummies, tow and straw, revealed a direct correlation between the deterioration of these materials and the microorganisms detected: strains related to *Chromohalobacter* species (*C.* *nigrandesensis*) and to *Nesterenkonia (N. halobia* and *N. halophila*) all showed cellulolytic activities. Species of these two genera have already been identified for the capability to degrade hemicelluloses (Govender et al. 2009; Kui et al. 2010; Prakash et al. 2009). The strains phylogenetically most related to an uncultured clone from the microbiome showed an individual variation, some being positive and some negative in the cellulolytic test (Table 1). The same was observed for the strains related to species of the genera *Staphylococcus* and *Halomonas*. Cellulase activities were already described for *Halomonas* sp. (Huang et al. 2010).

Although the hydrolytic properties of different halophilic bacteria were studied by other authors (Rohban et al. 2009; Sánchez-Porro et al. 2003), and offer a promising anticipation in a variety of biotechnological fields (Begemann et al. 2011; Setati 2010), the biodegradative assays developed in this investigation contribute to enlarge the armamentarium of microbiological media. The new media formulation enables to describe the proteolytic and cellulolytic activities of isolates using different substrates, not frequently used before, such as gelatine (skimmed milk is used often) and OBR-HEC, respectively. The crucial point during the development of these media regarded the salt precipitation. To avoid this phenomenon, different components of a specific medium were sterilized separately and then mixed together at a temperature of around 45 °C. When all the components in a medium were autoclaved together, the resulted agar plates did not allow a reliable recognition of biodegradation zones. Such novel media can be usefully utilized in testing the proteolytic and cellulolytic abilities of other strains isolated from other NaCl-extreme environments, and therefore can be of valuable help in studying the characteristics of several halophylic strains.

Concerning the observed discoloration phenomena, many of the isolated strains, especially those isolated from media containing higher NaCl concentrations, displayed a strong pigmentation, revealing a correlation between the pigmented isolated bacteria and the discoloration of the investigated materials. On sample W1, showing rosy discoloration, pigmented bacteria ranging from salmon–yellowish (strains W1-1a, W1-1b, W1-3, W1-4, W1-5, W1-6, W1-10 and W1-12), salmon (strains W1-9 and W1-14) to pink color (strain W1-16) were isolated. On sample W2, taken from a yellowish salt efflorescence, the isolated bacteria showed a pigmentation that ranged from beige–yellowish (strain W2-5), salmon–yellowish (strains W2-6 and W2-7) to yellow–orange colonies (strains W2-8, W2-9, W2-10 and W2-11). The bacteria isolated from sample PS, showing purple stains, showed to be more diverse concerning the pigmentation of the isolated bacteria, ranging from yellow (strains PS4 and PS6), salmon (PS22 and PS23) salmon–yellowish (PS7b), salmon–orange (PS7, PS10, and PS11) to dark pink (PS13, PS15, PS17, PS18, PS19a, PS20 and PS24), with the latter being dominant on the sample. Sample C3, showing rosy-orange discoloration, consisted of bacteria showing pigmentation ranging from yellow (C3-2, C3-10, C3-13), salmon (C3-3) to intense orange (C3-12, C3-14 and C3-15).

## Conclusions

The Capuchin Catacombs of Palermo provide a good example of a salt-attacked environment, offering a habitat for moderately and extremely halophilic microorganisms. The cultivable micro-biota inhabiting the Catacombs consists of a community highly adapted to their unique saline habitat. The predominant isolation of spore-forming bacteria, such as *Halobacillus* and *Bacillus* sp., can be explained by the fact that they can survive in unfavorable conditions: when these are again favorable, they are able to grow rapidly from their spores in the culture media. This fast growth can lead to an overestimation of the number of these microorganisms actually living on these substrates.

The two different PCR-based methods used for discrimination and clustering of the isolates, f-ITS and RAPD analyses, produced typical profiles, but also evidenced the different characters of these two molecular tools. The f-ITS method is suitable to cluster the isolates more at the species level, while the RAPD consents a subspecies typization.

The proteolytic and cellulolytic abilities screened through the use of plate assays showed that many of the strains isolated from the wall samples displayed proteolytic activities. In addition, many of the strains isolated from stuffing materials showed cellulolytic activities. Finally, results showed that many of the isolated microorganisms were pigmented, ranging from yellow to strong pink color and being directly related to the discoloration displayed by the materials.
